# Regulation of ovulation rate in mammals: contribution of sheep genetic models

**DOI:** 10.1186/1477-7827-4-20

**Published:** 2006-04-12

**Authors:** Stéphane Fabre, Alice Pierre, Philippe Mulsant, Loys Bodin, Elisa Di Pasquale, Luca Persani, Philippe Monget, Danielle Monniaux

**Affiliations:** 1 INRA, Physiologie de la Reproduction et des Comportements, UMR 6175 INRA-CNRS-Université de Tours-Haras Nationaux, 37380 Nouzilly, France; 2 INRA, Laboratoire de Génétique Cellulaire, BP 27, 31326 Castanet-Tolosan, France; 3 INRA, Station d'Amélioration Génétique des Animaux, BP 27, 31326 Castanet-Tolosan, France; 4 Institute of Endocrine Sciences, University of Milan, Istituto Auxologico Italiano IRCCS, Via Zucchi 18, 20095 Cusano Milanino (MI), Italy

## Abstract

Ovarian folliculogenesis in mammals from the constitution of primordial follicles up to ovulation is a reasonably well understood mechanism. Nevertheless, underlying mechanisms that determine the number of ovulating follicles were enigmatic until the identification of the fecundity genes affecting ovulation rate in sheep, bone morphogenetic protein-15 (BMP-15), growth and differentiation factor-9 (GDF-9) and BMP receptor-1B (BMPR-1B). In this review, we focus on the use of these sheep genetic models for understanding the role of the BMP system as an intra-ovarian regulator of follicular growth and maturation, and finally, ovulation rate.

## Introduction

The general mechanism of ovarian folliculogenesis in mammals is reasonably well understood, implying a complex endocrine dialog between the central nervous system and the ovary, and various intraovarian paracrine regulations. However, in female mammals, the underlying mechanisms that control the number of ovulating follicles in each oestrus cycle, i.e. the ovulation rate, are still poorly understood. Women, cattle, goats and ewes have generally one or two offspring, whereas other mammals, such as rodents, dogs or sows are highly prolific and produce four or more offspring. However, in sheep, a large range in litter size has been observed among different breeds and within breeds. Genetic studies in sheep have indicated that the ovulation rate and litter size can be genetically regulated either by a set of different genes each having a small effect as in the Romanov breeds [1], or alternatively by the action of single genes with major effect, named fecundity (*Fec*) genes (reviewed in [2]). In this respect, sheep has been considered as a model species to identify genes involved in mechanisms controlling ovulation rate. Thus, for the last past two decades, geneticists have created informative families for segregation studies and fine mapping for some of the major genes affecting ovulation rate. Concomitantly, physiologists have largely investigated endocrine regulations of the reproductive axis (hypothalamus-pituitary-ovary) in low, compared to high ovulation rate breeds. The common goal was to identify key genes and physiological regulations that determine ovulation rate in ovine species. The purpose of this review is to highlight the use and the study of ovine breeds with different prolificacies that have led to the identification of genes of the Bone Morphogenetic Protein (BMP) system with unexpected role in the control of ovarian follicular growth and ovulation rate.

## Identification of the fecundity genes affecting ovulation rate in sheep

### BMP-15 and GDF-9

Among the dozen of major genes presently known to affect ovulation rate in sheep [2], Inverdale and Hanna were the first mutations that have been identified [3]. The Inverdale gene (*FecX*) was identified in a flock of Romney sheep in New Zealand. Based on the inheritance pattern, it was predicted the presence of a major gene carried by the X chromosome [4]. One copy of the Inverdale allele (*FecX*^*I*^) increases ovulation rate by 0.8 and enhance both the number and LH sensitivity of antral follicles in ovaries [5]. In contrast, homozygous Inverdale ewes (*FecX*^*I*^*/FecX*^*I*^) are sterile with small underdeveloped ovaries containing follicles with no more than one layer of granulosa cells [6,7]. The *FecX*^*I *^allele corresponds to a single T to A transition at nucleotide position 896 in the cDNA coding for the Bone Morphogenetic Protein-15 (BMP-15), also known as Growth and Differentiation Factor-9b (GDF-9b). This mutation causes a non-conservative substitution of valine with aspartic acid at amino acid 299 of the unprocessed peptide (amino acid 31 of the mature protein, V31D, Table 1). Galloway et al. [3] also described the identification of the Hanna mutation (*FecX*^*H*^) showing the same inheritance pattern and phenotype as the Inverdale [8]. Interestingly, the *FecX*^*H *^allele corresponds also to a single mutation C to T at nucleotide position 871, in the BMP-15 coding sequence. The Hanna mutation introduces a premature stop at the amino acid position 291 of the unprocessed peptide (amino acid 23 in mature protein, Q23stop) leading likely to a loss of bioactivity of the BMP-15 protein produced by the *FecX*^*H *^allele.

**Table 1:** Identified major genes affecting ovulation rate in sheep

Name	Gene (Chromosome)	allele	Mutation pro/mature protein	Founder breed	Reference
Inverdale	BMP-15 (X)	*FecX*^*I*^	V299D/V31D	Romney	[3]
Hanna		*FecX*^*H*^	Q291stop/Q23stop	Romney	[3]
Belclare		*FecX*^*B*^	S367I/S99I	Belclare	[9]
Galway		*FecX*^*G*^	T239stop/no	Belclare, Cambridge	[9]
Lacaune X-linked		*FecX*^*L*^	C321Y/C53Y	Lacaune	[13]
High Fertility	GDF-9 (5)	*FecG*^*H*^	S395F/S77F	Belclare, Cambridge	[9]
Booroola	BMPR-1B (6)	*FecB*^*B*^	Q249R	Merino, Garole, Javanese, Hu, Han	[21–25]

GenBank accession numbers: BMP-15 (AH009593); GDF-9 (AF078545); BMPR-1B (AF312016)

More recently, two new mutations in the BMP-15 gene and one in the closely related GDF-9 gene have been described in Belclare and Cambridge sheep [9]. The additional mutations in BMP-15 named *FecX*^*G *^(Galway) and *FecX*^*B *^(Belclare) are a C to T and a G to T transitions at nucleotides 718 and 1100, respectively. *FecX*^*G *^causes a premature stop codon at amino acid 239 of the unprocessed protein (no mature protein) and *FecX*^*B *^substitutes a serine with an isoleucine at amino acid position 367 of the unprocessed peptide (amino acid 99 of the mature protein, S99I, Table 1). The ovarian phenotype in animals homozygous for these mutations in BMP-15 is indistinguishable from the Inverdale phenotype [10]. The mutation *FecG*^*H *^(High fertility) in the GDF-9 gene on chromosome 5 is a C to T transition at position 1184 of the cDNA substituting a serine for a phenylalanine at position 77 of the mature peptide (S77F, [9]). Even if *FecG*^*H*^*/FecG*^*H*^ewes are infertile, ovarian follicles develop to an abnormal type 5 early antral stage [10], differing from the phenotype of homozygous *FecX *carriers where follicles are blocked at the primary stage.

Finally, a new autosomal major gene affecting ovulation rate has been evidenced in the French Lacaune breed [11]. The *FecL *locus has been mapped on sheep chromosome 11 [12,13] and the fine mapping is in progress to identify the gene. However, in the Lacaune population, ewes with extremely high ovulation rate have been observed and it has been hypothesized that another mutation is segregating in this population. In fact, a new mutation, named *FecX*^*L*^, was recently identified in the BMP-15 gene. It corresponds to a G to A transition at nucleotide 1196 of the cDNA, replacing a cysteine with a tyrosine at position 53 of the mature protein (C53Y; [13]). *FecX*^*L*^*/FecX*^*L*^homozygous ewes present the same "streak" ovaries phenotype than Inverdale homozygous ewes, with follicles blocked at the primary stage (L. Bodin, S. Fabre, P. Monget, unpublished observation).

### BMPR-1B

Booroola was the first major gene that has been reported to increase ovulation rate [13,14]. The hyperprolific phenotype of the Booroola ewes appeared in a breeding of Australian Merino sheep. It is due to the action of a single autosomal gene (*FecB*), which influences the number of ovulations per estrous cycle. Ewes which are homozygous *FecB*^*B*^/*FecB*^*B*^, heterozygous *FecB*^*B*^*/FecB*^+ ^and non-carriers *FecB*^+^*/FecB*^+ ^of the *FecB*^*B *^Booroola mutation can be segregated on the basis of ovulation rate recording of 5 or more, 3 or 4 and 1 or 2, respectively [15,16]. This increase in ovulation rate of *FecB*^*B *^carriers is associated with a precocious maturation of a large number of antral follicles that ovulate at a smaller size than non-carrier follicles [17-19]. Moreover, the dynamics of final follicular growth is changed in *FecB*^*B *^carrier ewe since preovulatory follicles reach their maximal size early in the follicular phase and remain at a plateau until ovulation [20]. In 2001, three independent groups published simultaneously the mutation responsible for the hyperprolific phenotype of the Booroola ewes [21-23]. The *FecB*^*B *^allele corresponds to a single mutation in the coding sequence of the Bone Morphogenetic Protein Receptor-type 1B (BMPR-1B), also known as Activin-like kinase receptor-6 (ALK-6), on ovine chromosome 6 (Table 1). The G to A transition at nucleotide position 746 of the cDNA induces a non-conservative substitution of a glutamine with an arginine at position 249 of the protein (Q249R).

After identification of these mutations, several known prolific breeds have been screened for *FecX*^*I *^and *FecB*^*B*^. *FecX*^*I *^was not found in any of the breeds tested, but *FecB*^*B *^was present in the Indian Garole, Indonesian Javanese as well as Chinese Hu and Small-tailed Han breeds of sheep [24,25].

In conclusion, these observations point out the BMP-15 gene as a mutational "hot spot" in sheep with already 5 different mutations identified (Table 1). Together with the identification of the mutated GDF-9 and BMPR-1B genes, all belonging to same cellular signaling system, these results highlight the importance of the BMP peptides in the regulation of ovulation rate. Additionally, the fact that all these mutations in sheep are associated with increased ovulation rate without a drastic change in gonadotropins secretion [26] indicates that BMPs would act essentially as paracrine and/or autocrine regulators of ovarian follicular development and growth [27].

## The bone morphogenetic protein system

The BMP system belongs to the Transforming Growth Factor ß (TGFß) superfamily of growth factors and receptors, along with Growth and Differentiation Factors (GDF), activin/inhibin peptides, Anti-Mullerian Hormone (AMH) and the Myostatin protein. The BMP factors act through two subtypes of single transmembrane domain receptors with serine-threonine kinase activity. Alk-2, 3 and 6 act as type 1 receptors for BMP factors, whereas BMP receptor-2 (BMPR-2), Activin receptor-2 and 2B (ActR-2, ActR-2B) are type 2 receptors. Dimeric BMP molecules interact with heterotetrameric complexes of type 1 and type 2 receptors. Within each complex, the constitutive kinase activity of the type 2 receptor phosphorylates the type 1 receptor. Once phosphorylated, the type 1 receptor recruits and phosphorylates intracellular signaling molecules of the Smad proteins family. The phosphorylated BMP receptor-regulated Smads (Smad 1, 5, or 8) interact with the common Smad 4 and translocate to the nucleus where these hetero-complexes act as transcriptional factors or co-factors to regulate target genes expression (reviewed in [28]).

Within this general mechanism, it has been shown that *FecX*/BMP-15 exhibits the highest affinity for the *FecB*/BMPR-1B/Alk-6 and the BMPR-2 receptors, consistent with the activation of the BMP-specific Smad 1 signaling pathway [29]. In contrast, *FecG*/GDF-9 induces the phosphorylation and the activation of the TGFß/activin-specific Smad 2 signaling pathway by interacting with an unusual BMPR-2/Alk-5 receptor complex [30,31]. Thus, BMPR-1B could act as a potent receptor for BMP-15 but not GDF-9. However, GDF-9 and BMP-15 are likely to cooperate since they can form homodimers and heterodimers when produced in the same cell in culture [32].

## Expression of the BMP system in the sheep ovary

**Figure 1:**
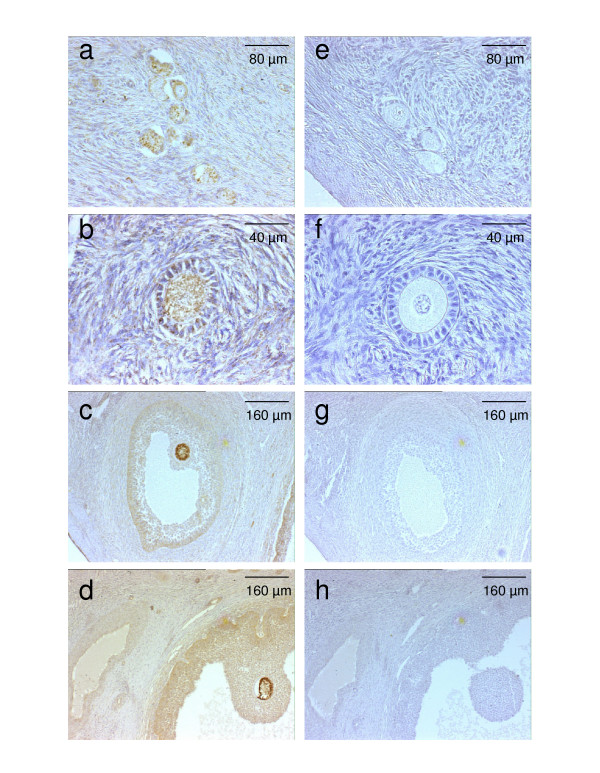
**Immunostaining for BMPR-1B in sheep ovary**. Photomicrographs of primordial (a, e), primary (b, f), small antral (c, g) and large antral follicles (d, h). The photomicrographs (a-d) correspond to immunostaining with anti-BMPR-1B rabbit polyclonal antibody (1/1000; R&D Systems). The photomicrographs (e-h) correspond to immunostaining with control rabbit IgG.

The expression of the three fecundity genes has been observed in the sheep ovary. Oocytes are the only sources of GDF-9 and BMP-15 [33]. The BMP receptor BMPR-1B is expressed by granulosa cells and oocytes from the primary to the late antral follicle stages (Fig. 1), and to a lesser extent, by the theca layer of ovine and bovine antral follicles [23,34,35]. These three genes are not the only genes of the BMP signaling system expressed in the ovary. Among about twenty molecules of the BMP/GDF subgroup of TGFß superfamily [36], mRNA for BMP-2, BMP-4, BMP-6 and BMP-7 have been detected in sheep ovary [37]. BMP-4 and BMP-7 mRNA are expressed by granulosa and theca cells, whereas BMP-2 mRNA is only expressed by granulosa cells and BMP-6 mRNA by oocytes [37]. In bovine antral follicles, Glister et al. [35] have detected the presence of BMP-4 and BMP-7 proteins in theca cells whereas granulosa cells and oocyte selectively express BMP-6. Among the family of BMP receptors, the BMPR-1A/Alk-3 receptor, closely related to BMPR-1B, and the BMPR-2 receptor are expressed mainly in the granulosa cells of primary to late antral follicles in sheep ovary [34]. The presence of ActR-1A, ActR-2 and ActR-2B has also been detected in bovine antral follicles [35]. Moreover, the intracellular downstream signaling Smad 1 protein has been detected in ovine and bovine granulosa cells [35,38], and Smad 2 has been detected in bovine granulosa and theca cells [35,39]. The nature and localization of these BMP family members within the ruminant ovary seem similar to those described in rodents [40,41].

Recently, expression of the three *Fec *genes, BMP-15, GDF-9 and BMPR-1B, has been detected by RT-PCR analysis during ovarian development in fetal sheep [42]. Among the three genes, BMPR-1B is the most precociously expressed, as early as 25 days post-coïtum (dpc) before the gonadal sex differentiation. BMPR-1B expression significantly increases at 56 dpc at the time of germinal cell meiosis. GDF-9 transcripts are detected at 56 dpc and fully expressed at 75 dpc at the time of primordial follicles formation. Accordingly, the expression of GDF-9 has been observed in oocytes of type 1-1a follicles [10,33,43]. BMP-15 transcripts are detected only from 94 dpc that corresponds to the appearance of the first growing follicles. This is in agreement with the observation of BMP-15 expression in oocytes from primary type 2 follicles [3,10]. Interestingly, the absence of expression of GDF-9 and BMP-15, and the slight expression of BMPR-1B in the developing testis compared to ovary [42] may be in favor of a lower incidence of the BMP system in testicular function, in agreement with the absence of an altered reproductive phenotype in male carriers of the mutated *Fec *genes. The transcripts of BMP-4, BMPR-1A, BMPR-2, Smad 1, 4 and 5 are also detected as early as 32 dpc in the developing ovine ovary (B. Mandon-Pépin, S. Fabre, unpublished data).

Collectively, these data indicate that a complete BMP signaling system is present in ruminant ovaries from fetal to adulthood stage. Thus, it may exert autocrine and paracrine actions in the formation and the development of ovarian follicles. Numerous BMP growth factors are expressed specifically or mainly by the oocyte (BMP-15, GDF-9 and BMP-6), the others being expressed by the somatic follicular cells (BMP-2, BMP-4 and BMP-7). All the cellular compartments of the follicle, especially the granulosa cells, are equipped with BMP receptors and then, are targets of BMP biological action.

## The role of sheep fecundity genes in folliculogenesis

### FecX/BMP-15

**Figure 2:**
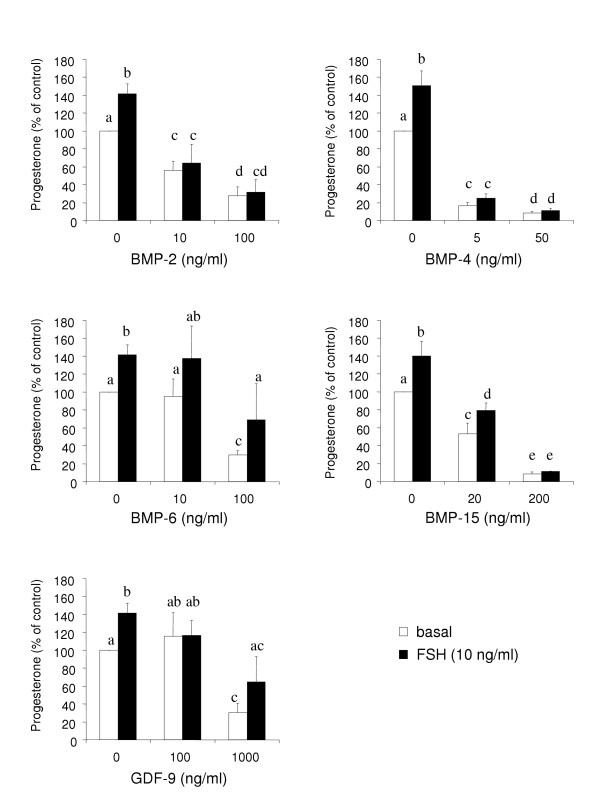
**Progesterone secretion by sheep granulosa cells**. Granulosa cells from small antral follicles of 1–3 mm in diameter were cultured for 96 h in serum free media in the presence or absence of FSH (10ng/ml), in combination with varying concentrations of recombinant human BMP-2, BMP-4, BMP-6 (each from R&D Systems), BMP-15 [46] or rat GDF-9 [76]. Each combination of treatments was tested in triplicate in each of 4 independent experiments. Results represent progesterone secretion by 50,000 granulosa cells between 48 h and 96 h of culture. Data are expressed as percentages (mean ± SEM) of the amount of progesterone secreted by cells cultured in control condition. Different letters denote significant difference (p < 0.05).

Recombinant BMP-15 increases the proliferation of granulosa cells from rat [44,45] and human [46]. Additionally, in granulosa cells, BMP-15 potently stimulated mRNA encoding Kit ligand a factor that is necessary for oocyte growth in preantral follicles. Thus, both BMP-15 and Kit ligand play important roles in early follicular growth [47]. Furthermore, BMP-15 is able to modulate steroidogenesis of granulosa cells. Indeed, in the rat, BMP-15 selectively modulates the biological effects of FSH on granulosa cells by inhibiting FSH-induced progesterone production without affecting FSH-induced oestradiol synthesis [44]. The underlying mechanism implies the down-regulation of the FSH receptor [48] leading to the suppression of mRNA accumulation of numerous FSH-dependent genes such as steroidogenic acute regulatory protein (StAR), P450 side chain cleavage enzyme (P450scc), 3ß-hydroxysteroid deshydrogenase (3ß-HSD), LH receptor and inhibin/activin subunits [49]. In the sheep, BMP-15 also increases proliferation rate [50] and suppresses basal and FSH-induced progesterone secretion by granulosa cells from small antral follicles (Fig. 2).

### FecG/GDF-9

Like BMP-15, the closely related oocyte growth factor GDF-9 is a potent stimulator of granulosa cells proliferation in the rat [45,51]. However, some effects on rodent granulosa cells are different from those of BMP-15, likely due to the use of different signaling pathways. Firstly, GDF-9 has been shown to inhibit Kit ligand expression [52,53]. Secondly, GDF-9 inhibits both FSH-induced progesterone and oestradiol production [51] while it enhances basal progesterone secretion associated with up-regulation of the StAR gene [52], acting via a prostaglandin E2 pathway [54]. Moreover, GDF-9 decreases LH receptor mRNA synthesis and also exerts specific effects on cumulus cells since it induces cumulus expansion and enhances hyaluronan synthase 2 (HAS2) and cyclooxygenase 2 (COX-2) expression [52]. Additionally, GDF-9 is able to stimulate androgen biosynthesis by thecal cells in the rat [55]. Even if BMP-15 and GDF-9 do not use the same signaling pathway, as said above, these two factors were shown to co-operate in enhancing granulosa cell proliferation, increasing inhibin production and suppressing progesterone secretion [45].

**Figure 3:**
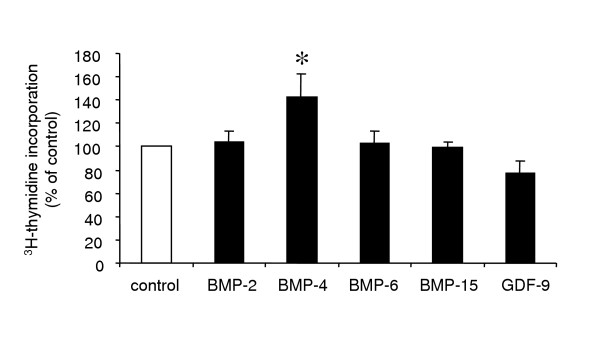
**^3^H-thymidine incorporation into sheep granulosa cells**. Granulosa cells from small antral follicles of 1–3 mm in diameter were incubated with ^3^H-thymidine for 2 h after 48 h of culture in the presence of 3% fetal ovine serum with or without recombinant human BMP-2 (100 ng/ml), BMP-4 (50 ng/ml), BMP-6 (100ng/ml), BMP-15 (200 ng/ml) or rat GDF-9 (1000 ng/ml). Results represent the labeling index (percentage of ^3^H-thymidine-labelled cells). Data are expressed as percentages (mean ± SEM) of the labeling index of cells cultured in control condition. Asterisk indicates significant difference (p < 0.05) compared with control.

In the sheep, we have observed a potent action of GDF-9 on suppressing both basal and FSH-stimulated progesterone production by ovine granulosa cells from small antral follicles (Fig. 2), with proliferation being unaffected (Fig. 3).

### FecB/BMPR-1B

BMPR-1B has been described as a potent receptor for various BMP factors [56]. Among them, BMP-15 but also BMP-2, -4, -6 and BMP-7 are all present within the ovary. Then, to understand the role of BMPR-1B in the ovary, it is important to establish the biological effect of its potential ligands on granulosa cells, theca cells and oocytes, all expressing BMPR-1B. In sheep granulosa cells from small antral follicles, BMP-2, 4, and 6 all inhibit both basal and FSH-stimulated progesterone production (Fig. 2). The inhibitory effect of BMP-4 on progesterone secretion is associated with a decrease in expression of StAR, 3ß-HSD and P450scc genes at the mRNA and protein levels [38,57]. The underlying mechanism implies a decrease in FSH-induced cAMP production as well as a decrease in cAMP biological effects possibly on Steroidogenic factor-1 (SF-1) transcriptional activity, which is inhibited by Smad 1 [38]. In contrast, BMP-2, 4 and 6 enhance gonadotropin-induced oestradiol production [34,58]. The same effects, negative on progesterone and positive on oestradiol, have been observed for BMP-4, 6 and 7 using bovine granulosa cells in basal and Insulin-like growth factor-I (IGF-I)-stimulated conditions [35]. In addition, BMP-4 inhibits LH-dependent production of progesterone by granulosa cells from preovulatory follicles (A. Pierre and S. Fabre, unpublished data). Among the different BMP factors tested, only BMP-4 appears to increase ovine granulosa cell proliferation (Fig. 3) [57]. In the rat, BMP-7 represents also a potent stimulator of granulosa cells proliferation [59]. Additionally, BMP-2, 4 and 6 are able to decrease LH-induced androstenedione production by ovine theca cells [58]. Accordingly, BMP-4, 6 and 7 suppress basal and LH-induced androgen production by bovine theca cells [39]. These factors are also potent stimulators of theca cell number and proliferation [39,58].

Steroids are not the only secretion products of follicular cells that are affected by the action of BMPR-1B ligands. Indeed, BMP-2 enhances inhibin-B production in human granulosa-luteal cells [60] and inhibin-A secretion by ovine granulosa cells [34]. Moreover, BMP-4, 6 and 7 have been shown to increase inhibin-A, activin-A and follistatin production by bovine granulosa cells [35].

The oocyte could be considered as a target cell for BMPR-1B ligands. However, BMP-2 and BMP-4 do not affect oocyte nuclear maturation, cumulus cell expansion, or blastocyst formation following IVF in the bovine species [61].

Collectively, these results indicate that fecundity genes are implicated in mechanisms regulating granulosa and theca cells proliferation and differentiation. Along the folliculogenesis process, *FecX*, *FecG *and *FecB *genes likely control the early steps when follicular growth is closely linked to granulosa cell proliferation. At later stages of follicular development, in antral follicles, *Fec *genes would likely modulate the differentiative effect of FSH, and possibly IGF-I, on follicular cells. Finally, in gonadotropin-dependent large antral follicles, *Fec *genes control follicular cells differentiation and exert a dramatic negative action on FSH and LH-dependent progesterone production. Thereby, they might be implicated in delaying the luteinization process in follicular cells before the time when ovulation and luteinization are triggered by the preovulatory gonadotropin surge.

## How do mutations in sheep fecundity genes affect the ovulation rate?

To answer this question, one has to know the functional consequences of the mutations in the normal biological activity of proteins produced by fecundity genes. Obviously, in the case of the *FecX*/BMP-15 gene, *FecX*^*H *^and *FecX*^*G *^create STOP codon and then would impair the production of biologically active mature BMP-15. Interestingly, the resulting ovarian phenotype is indistinguishable from the phenotype observed with the other three single mutations *FecX*^*I*^, *FecX*^*B *^and *FecX*^*L*^, likely enabling the complete peptide production as described for *FecX*^*I *^and *FecX*^*B *^[32,62]. Thus, these mutations, as well as *FecX*^*H *^and *FecX*^*G*^, can be considered as complete "loss of function" mutations for BMP-15. The precise molecular mechanism by which these mutations impair BMP-15 activity is still unclear. It may affect either the formation of BMP-15 homodimers [3] or the efficiency of the processing and secretion of homodimers or their heterodimerization with GDF-9 [32]. The *FecB*^*B*^, Q249R mutation in BMPR-1B, has also been hypothesized as a partial "loss of function" mutation [21,57]. Indeed, using a BMP-specific luciferase reporter in transfected HEK-293 cells, the presence of the Q249R mutation in BMPR-1B is associated with a loss of responsiveness to BMP-4 [57]. This loss in receptor activity is also illustrated by the fact that granulosa cells from homozygous *FecB*^*B *^carrier ewes are less sensitive to the action of BMP-4 on proliferation and inhibition of progesterone production than those from non-carrier ewes [21,57].

**Figure 4:**
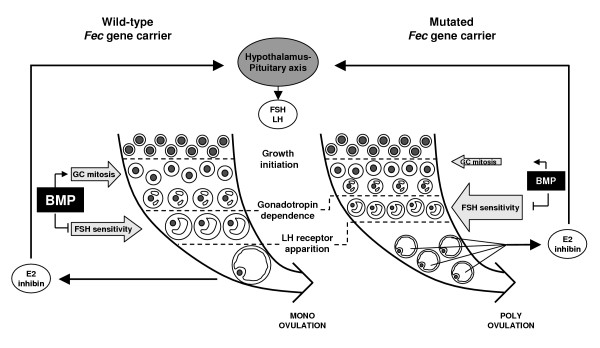
**Schematic representation of the effects of a mutation in fecundity *(Fec) *gene on folliculogenesis and ovulation rate in sheep**. The reduced activity of the BMP signaling system in the ovary of mutated *Fec *gene carrier (right) compared to non-carrier (left) ewes leads to decrease both the positive action of BMP on granulosa cell (GC) mitosis and its inhibiting action on FSH sensitivity. The consequence is the presence of smaller antral follicles with a reduced number of granulosa cells exhibiting a higher FSH sensitivity, leading to an advance in follicular maturation as attested by precocious LH receptor expression. The smaller matured follicles present in mutated *Fec *gene carrier each produce reduced amounts of oestradiol (E2) and inhibin, but altogether they produce the same amounts than one larger wild-type follicle. Consequently, the same endocrine dialog can establish between the ovaries and the central nervous system of both genotypes that leads to the selection and ovulation of numerous smaller follicles in mutated *Fec *gene carrier.

Thus, in sheep, it seems that a decrease in BMP system activity leads to an increase in ovulation rate. Based on this concept and the known role of BMP molecules during folliculogenesis, a mechanism can be proposed to explain the increase in ovulation rate due to "loss of function" mutations in the BMP system (Fig. 4). Firstly, mutations in *Fec *genes would likely impair the proliferative action of BMPs from the first steps of folliculogenesis onwards. The final consequence is the presence of follicles with a lower number of granulosa cells in ovaries from mutated *Fec *carrier ewes [63,64]. Secondly, in the presence of loss of function mutations, the BMP inhibiting action on the FSH pathway in granulosa cells is decreased, enabling a higher FSH sensitivity [5,65] and an enhanced expression of FSH-dependent markers of differentiation such as steroidogenic enzymes genes, inhibin/activin subunits and LH receptor in granulosa cells of antral follicles [63]. Accordingly, these markers of granulosa cells differentiation appear in follicles of smaller size in mutated *Fec *gene carriers compared to non-carriers. The increased sensitivity to gonadotropins of these follicles promotes their selection and maintenance when circulating concentration of FSH is decreasing during the follicular phase. In mutated *Fec *gene carriers, each of these selected follicles contains a reduced number of granulosa cells and produces lower amounts of oestradiol and inhibin, but altogether, these follicles produce the same amounts than fewer wild-type follicles [5,26,63]. It is likely that the positive feedback of oestradiol on GnRH secretion is triggered using the same oestradiol threshold in both carriers and non-carriers of mutated *Fec *genes, allowing ovulation and luteinization of numerous mature LH-responsive follicles in mutated *Fec *gene carriers.

## Sheep fecundity gene as fecundity genes for other mammals

As a basis for comparison, targeted invalidation in mouse has been generated for the identified fecundity genes in sheep. Unlike mutated *FecX*/BMP-15 homozygous ewes, Bmp15 null female mice (Bmp15^-/-^) are fertile but exhibit a slight decrease in ovulation rate with minimal ovarian histopathological defects [66]. In contrast, Gdf9^-/- ^null female mice are sterile [67]. Ovarian folliculogenesis of Gdf9^-/- ^mice is blocked at the primary follicle stage mimicking surprisingly the ovarian phenotype of the homozygous mutated *FecX*/BMP-15 sheep. However, it differs from the phenotype of the homozygous mutated *FecG*/GDF-9 sheep in which the follicular development is stopped at the small antral stage [9,10]. Altogether, these results could indicate that Gdf9 in mouse and BMP-15 in sheep play the same crucial mitogenic role in the primary-secondary follicle transition. Thus, Bmp15 is dispensable and Gdf9 obligatory for normal folliculogenesis and ovulation in mouse. In contrast, both GDF-9 and BMP-15 are essential in the sheep as attested by the sterility of both homozygous mutated *FecX *and *FecG *ewes [3,9] and wild-type ewes immunized against GDF-9 and BMP-15 [33,68]. Mice invalidated for Bmpr-1b gene have also been established [69]. In striking contrast with *FecB*/BMPR-1B mutated hyperprolific sheep, bmpr-1b^-/- ^null mice are infertile due to cumulus expansion and postovulatory defects, but without alteration of follicle recruitment and maturation. These discrepancies might be due to species differences, and/or to the nature of the alteration (point mutation vs. deletion). Nevertheless, it should be noted that neither Bmp15 nor Gdf9 or Bmpr-1b invalidated mouse models exhibit at heterozygous or homozygous state an increase in ovulation rate as observed in sheep.

From the discovery of sheep fecundity genes and their role in regulating ovarian folliculogenesis, several research groups have focused on BMP-15 and GDF-9 alterations associated with human ovarian pathologies such as premature ovarian failure (POF) or polycystic ovary syndrome (PCO) [46,70,71]. At that time, a decreased expression of GDF-9 in oocytes of women with PCO [71] and an inactivating mutation in the BMP-15 proregion (Y235C) associated with POF have been described [46]. For the future, emerging systematic sequencing programs of these two genes in POF and PCO patients are operative with the perspective to discover new mutations.

## BMP dose dependent effect, from sterility to polyovulation

**Figure 5:**
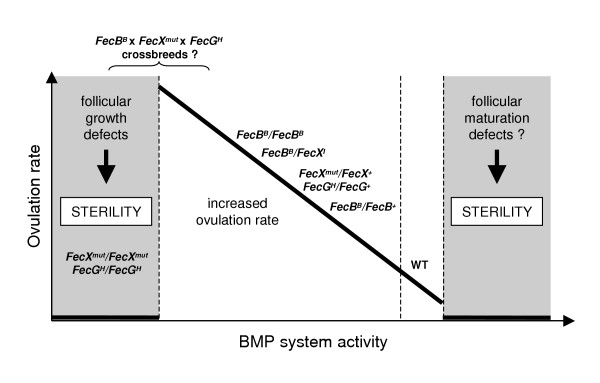
**Relationship between BMP system activity and ovulation rate in sheep: working hypothesis**. Increased ovulation rate is associated with loss of function mutations in the BMP system with additive or multiplicative effects of the mutated *FecX*, *FecG *or *FecB *genes. Thus, the relationship is based on the concept that ''the more the BMP system activity is reduced, the more the ovulation rate increases''. However, when the level of BMP activity is too low, folliculogenesis is blocked at early stages, leading to sterility as observed in homozygous *FecX*^*mut *^(mut for alleles I, H, B, G or L) or homozygous *FecG*^*H *^ewes. At the opposite, over-activity of the BMP system may lead to anovulation, then sterility, due to intensive inhibition of gonadotropin action. It is also hypothesized that crossbreeding between the 3 known mutated *Fec *genes might lead either to over-increased ovulation rate or to sterility.

As shown previously, the Booroola BMPR-1B mutation has additive effects on ovulation rate. Moreover, crossbreeding of sheep carrying one copy of a *FecX*/BMP-15 mutation with those carrying the *FecB*/BMPR-1B mutation exhibits a multiplicative effect on ovulation rate. Finally, one copy of a *FecX*/BMP-15 mutation together with one copy of the *FecG*/GDF-9 mutation suggests that the effect of the two genes is additive on ovulation rate (reviewed in [2]). Thus, it appears that in sheep, the more the BMP system activity is reduced, the more the ovulation rate increases (Fig. 5). At both extremities of this control might appear ovarian dysfunction and then sterility. In one side, total loss of BMP-15 or GDF-9 leads to arrest in folliculogenesis, while on the other side, one could hypothesize that hyperactivity of the BMP system might lead to non-fully mature anovulatory follicles (Fig. 5). Accordingly, *in vivo *treatment with BMP-7 markedly decreases ovulation rate in mice [59]. Whether this BMP "tune" in ovine species is the general mechanism that controls ovulation rate among mammals remains to be determined. In this way, the BMP-15 example is very interesting. Indeed, it appears that in the mono-ovulating human species, two functional copies of BMP-15 are required, since the presence of a heterozygous mutation is sufficient for ovarian failure. In ovine species, with a large ovulation rate ranging, only one copy is sufficient, homozygous mutation leading to ovarian function arrest. Finally, in a poly-ovulating species such as the mouse, with ovulation rate about 10, BMP-15 is dispensable. However, BMP-15 is present and expressed in oocytes of these three species. As a possible explicative mechanism, Hashimoto et al. [72] have shown that the mouse BMP-15 peptide is much less efficiently processed and secreted than the human peptide by the same transfected cells. Whether this deficient processing occurs also with the porcine BMP-15 will be of great interest in this highly poly-ovulating species where a functional BMP system in the ovary has also been demonstrated [73,74].

Linked to this BMP dose-dependence concept, ovaries express a wide variety of genes of the BMP system, raising the question of the redundancy and the relative importance of each of them along the folliculogenesis process within a given species and the regulation of ovulation quota between species with different ovulation rates. The answers to these questions are part of future field of investigation for the years to come.

## Conclusion

Since 1980, when Piper and Bindon [14] proposed that the exceptional fecundity of the Booroola Merino sheep may in part result from the action of a single major gene, the use of sheep with genetic mutation affecting ovulation rate has provided exceptional tools in the field of female reproductive biology. With the identification of the nature and the physiological role of fecundity genes, BMP-15, GDF-9 and BMPR-1B, two new major concepts were born. First, the BMP system, originally described as inducer of osteogenesis and chondrogenesis and implicated in early developmental events, now represents a key system as gonadotropins or IGFs systems, in the control of ovarian folliculogenesis and ovulation rate. Second, the oocyte, acting through specific secreted proteins, is not only implicated in follicular growth but also in the control of the number of ovulating follicles [75].

Numerous other major genes affecting ovulation rate in sheep remain to be identified [2]. Their identification may confirm the exceptional role of the BMP system or may point at genes independent of BMP signaling with main effects at the level of the ovary or the central nervous system or both.
